# Dexmedetomidine reduces the incidence of fentanyl-induced cough: A double-blind, randomized, and placebo-controlled study

**DOI:** 10.3109/03009734.2011.629749

**Published:** 2012-02-15

**Authors:** Liang He, Jun-Mei Xu, Ru-Ping Dai

**Affiliations:** Department of Anesthesia, The Second Xiangya Hospital of Central South University, Changsha, China

**Keywords:** Cough, dexmedetomidine, fentanyl, opioid-induced muscular rigidity

## Abstract

**Objectives.:**

The incidence of fentanyl-induced cough (FIC) during induction of general anesthesia varies around 40% and is undesirable. It increases intracranial, intraocular, and intra-abdominal pressures. This prospective, randomized, double-blind, placebo-controlled study evaluated the effect of dexmedetomidine (DEX) pretreatment on the incidence and severity of FIC.

**Methods.:**

Altogether 300 patients undergoing elective surgical procedures were randomly allocated into three groups (I, II, III; *n* = 100) and administered intravenously, over 10 min, 10 mL isotonic saline, DEX 0.5 μg/kg in 10 mL isotonic saline, or DEX 1 μg/kg in 10 mL isotonic saline, respectively. All groups subsequently received a fentanyl (4.0 μg/kg) intravenous push. The incidence and severity of cough were recorded for 1 min after fentanyl administration.

**Results.:**

The incidence of FIC was 61%, 40%, and 18% in groups I, II, and III, respectively (*P* < 0.05 for treatment groups II and III versus control group I). There was no significant difference in the severity or onset time of cough, or hemodynamic variables, among the three groups.

**Conclusions.:**

Intravenous DEX (0.5 μg/kg or 1 μg/kg) immediately before the administration of intravenous fentanyl (4.0 μg/kg) significantly reduced the incidence of FIC.

## Introduction

Fentanyl is commonly administered during general anesthetic induction. Fentanyl-induced cough (FIC) is undesirable as it increases intracranial, intraocular, and intra-abdominal pressures (1). The incidence of FIC varies from 18% to 65% (1–4). The mechanism of FIC is unclear, and various drugs and techniques are used to reduce its incidence.

Clonidine was found effective in reducing the incidence of FIC (5). Dexmedetomidine (DEX), another but more specific α-2 adrenoreceptor agonist, may also be a useful suppressant for FIC.

We designed a double-blinded, randomized, placebo-controlled study to test our hypothesis that DEX decreases the incidence of FIC.

## Materials and methods

### Patient population

The Ethics Committee of the Second Xiangya Hospital of Central South University approved this study, and all patients gave written informed consent. The study population consisted of 300 patients of both genders, aged 18 to 60 years, classified as American Society of Anesthesiologists (ASA) physical status I or II and scheduled for elective surgery under general anesthesia. Exclusion criteria were: body-weight more than 20% above ideal body-weight (on the basis of body mass index), impaired kidney or liver function, presence of a gastric tube, a history of asthma, chronic cough, smoking, upper respiratory tract infection in the previous 2 weeks, or angiotensin-converting enzyme inhibitor treatments, bronchodilators, or steroids in the previous 2 weeks.

### Anesthesia induction and data collection

None of the patients received any premedication. Before being taken to the operating room, a 20-gauge cannula was inserted into the dorsum of the patient’s hand and connected to a T-connector for drug administration. Upon arrival, standard ASA monitors were attached, including non-invasive arterial pressure, electrocardiography, and pulse oximetry.

Patients were randomly allocated to three groups (I, II, III; *n* = 100 each) using a computer-generated table of random numbers. All patients were given oxygen via a face mask (2 liters per min). The patients in group I (the control) were given 10 mL isotonic saline intravenously (IV) over 10 min. Patients in groups II and III received DEX 0.5 μg/kg or 1.0 μg/kg, respectively, mixed with isotonic saline to a final volume of 10 mL, over 10 min. A fentanyl bolus (4.0 μg/kg) was then injected into all patients in less than 2 s.

Beginning immediately, an observer blinded to the treatment group noted during the next 60 s the incidence and severity of cough. Any episode of cough within 60 s of fentanyl administration was classified as FIC, and the severity was graded based on the number of coughs (mild, 1–2; moderate, 3–4; and severe, ≥5). Mean arterial pressure (MAP) and heart rate (HR) were recorded immediately before injection (T_0_) and every 2 min thereafter for 10 min (T_1–5_).

### Statistical analyses

For the design of this study, an estimation of the required minimum sample size was determined based on a previous report of a cough incidence of 40% after an IV bolus of fentanyl (1) and an assumption that a pretreatment with DEX would cause a 50% reduction in the incidence of coughing. With a probability of making a type I error (i.e. significance level, α) = 0.05 and the probability of making a type II error (accepting a null hypothesis that is false, β) = 0.20, we were required to enroll at least 91 patients in each group; we recruited 100 patients in each group.

Data are expressed as number, proportion, percentage, or mean ± standard deviation. Statistical analyses were performed using Statistical Product for Social Sciences (SPSS) software 13.0. The frequencies of cough and the proportions of gender and ASA class were compared using the chi-square test or Fisher’s exact test with Bonferroni correction. One-way analysis of variance (ANOVA) was used to compare the ages and weights among the three groups. Repeated ANOVA was used to compare the means of continuous data of all three groups. If group differences were found by ANOVA to be significant, they were further analyzed using Tukey’s *Post-hoc* test. A probability (*P*) value < 0.05 was considered statistically significant.

## Results

### Demographic characteristics

All patients completed the study. There were no statistically significant differences among the three groups with regard to age, weight, gender, or ASA class (Table I).

**Table I.** Demographics of the control (I) and treatment (II, III) groups (*n* = 100, each)^a^.

Demographics	Group I	Group II	Group III
Age (y)	38.1 ± 16.6	41.2 ± 14.7	39.4 ± 17.3
Gender (M/F), *n*	62/38	59/41	65/35
Weight (kg)	56.4 ± 11.6	58.3 ± 10.9	55.9 ± 12.1
ASA (I/II), *n*	72/28	75/25	69/31

^a^All patients completed the present study. There were no statistically significant differences among the three groups with regard to age, weight, gender, or ASA class.

### Incidence and severity of FIC

The incidence of FIC was 61% in group I, 40% in group II, and 18% in group III. Groups II and III had a significantly lower incidence than group I (*P* < 0.05). The incidence of FIC in group III was significantly lower than in group II (*P* < 0.05). However, there was no significant difference in the severity or onset time of cough among the three groups (Table II).

**Table II.** Incidence, severity, and onset time in seconds of fentanyl-induced cough (FIC) in the control (I) and treatment (II, III) groups (*n* = 100, each).

FIC	Group I	Group II	Group III
Incidence (%)	61	40^a^	18^a^,^b^
Severity			
Mild	30	19	10
Moderate	23	16	6
Severe	8	5	2
Onset (s)	23.1 ± 4.6	26.1 ± 5.0	29.2 ± 4.1

^a^ *P* < 0.05, groups II and III versus group I.

^b^ *P* < 0.05, group III versus group II.

There were also no significant differences in the hemodynamics data among the three groups (Table III).

**Table III.** Hemodynamics data of the control (I) and treatment (II, III) groups (*n* = 100, each)^a^.

	Group	T_0_	T_1_	T_2_	T_3_	T_4_	T_5_
MAP (mmHg)	I	86.7 ± 11.8	85.2 ± 12.1	87.2 ± 11.3	83.5 ± 11.6	86.1 ± 10.9	85.3 ± 10.7
	II	83.2 ± 11.2	89.2 ± 12.3	82.1 ± 10.9	82.3 ± 10.2	80.5 ± 11.6	79.4 ± 10.1
	III	85.1 ± 10.1	92.3 ± 11.8	83.4 ± 10.4	80.1 ± 9.80	78.2 ± 12.1	77.9 ± 10.5
HR (bpm)	I	77.6 ± 14.5	78.2 ± 14.1	79.8 ± 12.6	76.4 ± 13.9	77.3 ± 14.7	75.1 ± 13.5
	II	79.2 ± 15.0	77.5 ± 14.9	75.7 ± 13.1	73.4 ± 14.3	73.0 ± 13.8	2.6 ± 13.5
	III	78.6 ± 14.1	75.3 ± 13.2	74.1 ± 13.5	72.6 ± 12.8	71.4 ± 13.6	71.2 ± 12.3

^a^There were no significant differences among the groups for either MAP or HR at any time point after DEX injection.

T_0–5_ = immediately before DEX injection, and at 2-min intervals, respectively, for 10 min total. DEX = dexmedetomidine; HR = heart rate; MAP = mean arterial pressure.

## Discussion

This study demonstrated that pretreatment with DEX, 0.5 and 1 μg/kg, reduced the incidence of FIC from 61% (in the untreated control) to 40% and 18%, respectively. However, the severity and onset time of cough was unaffected, and no significant hemodynamic changes were observed following DEX administration.

DEX, an α_2_-adrenoreceptor agonist, is widely used in the anesthetic setting and in intensive care (6–13). This is the first report on the effect of DEX on FIC, although the less specific α_2_-adrenoreceptor agonist clonidine has been demonstrated to reduce the incidence of FIC (5).

Many mechanisms have been proposed to explain FIC. These include the stimulation of vagal C-fiber (J or juxtacapillary) receptors, citric acid stimulating C-fibers in the airway, the release of histamine from lung mast cells, sudden adduction of the vocal cords or supraglottic obstruction by soft tissue, deformation of the tracheobronchial wall stimulating irritant receptors that lead to reflex bronchoconstriction and cough, and the release of neuropeptides from prejunctional μ-opioid receptors (2,3,14–18), Fentanyl-induced muscle rigidity is another important causal factor. The α_2_-adrenoreceptor agonists’ ability to reverse opioid-induced muscular rigidity has been demonstrated in rats (19). It is possible that α_2_-adrenoreceptor agonists reduce the incidence of FIC via reversal of fentanyl-induced muscular rigidity and not through sedation (20). The investigation by Horng et al. (5) demonstrated that clonidine could suppress FIC in humans. Thus, we suggest in this study that the effect of DEX on FIC occurs via reversal of fentanyl-induced muscular rigidity.

Many physical methods and drugs have been reported to prevent FIC, including propofol, lidocaine, and ephedrine (1,3,5,14,17,21,22). All reported methods have variable effectiveness and potential side effects. The efficacy of physical methods for reducing FIC remains controversial. The investigation by Yu et al. (4) demonstrated that dilution of fentanyl combined with a prolonged injection time could eliminate FIC. However, according to Schäpermeier and Hopf’s study (23), FIC does not depend on injection speed. A huffing maneuver was reported as a useful way to prevent FIC (24), but some patients who receive midazolam or propofol during induction of general anesthesia cannot use this maneuver.

Although the above-mentioned medications could reduce the incidence of coughing, some unexpected side-effects may occur during drug administration, such as malignant arrhythmia, hypotension, and hypertension. Pretreatment with lidocaine can augment the cardiovascular depression of induction agents (25). Intravenous ephedrine before fentanyl injection can be contraindicated in patients with coronary artery disease or moderate to severe hypertension (24). Using high doses of propofol can be associated with a high incidence of hypotension (22). Pretreatment with clonidine is also associated with respiratory depression, drowsiness, and severe hypotension (5).

Pretreatment with DEX has been reported to cause significant hemodynamic changes (26). However, our study demonstrates that DEX in doses of 0.5 μg/kg or 1 μg/kg can be safely used preoperatively, with stable hemodynamics. A report of Koroglu et al. showed that a high dose of DEX (a bolus of 2–3 μg/kg over 10 min or infusion of 1.5–3.0 μg/kg·h) in children undergoing MRI produced bradycardia in 16% of these patients. However, the MAP and oxygen saturation remained within normal range, and no adverse sequelae were observed and no specific treatment was required (13).

A limitation of this study was that a dose-response experiment was not performed to determine the optimal dose of DEX that produces the maximum depression of FIC without causing side effects. However, our choice of doses was based on the DEX dose used in premedication (27). DEX is used as a premedicant in doses of 0.5 to 1 μg/kg because of its sedative and anesthetic-sparing effects, as well as attenuating airway/circulatory reflexes during anesthesia (11). We chose the doses 0.5 μg/kg and 1 μg/kg and achieved satisfactory effects, whilst maintaining hemodynamic stability. Further studies are warranted to determine the optimum dose of DEX that will suppress FIC without causing side effects.

In conclusion, intravenous DEX (0.5 μg/kg or 1 μg/kg) given immediately before the administration of IV fentanyl (4.0 μg/kg) is a convenient way to reduce the incidence of FIC.
